# Pediatric CLIPPERS: Expanding the Neuroimaging Spectrum of a Rare Steroid-Responsive Neuroinflammatory Syndrome—A Systematic Review

**DOI:** 10.3390/brainsci16080790

**Published:** 2026-07-27

**Authors:** Alessio Leoncini, Alessandro Fazio, Vincenzo Sortino, Manuela Lo Bianco, Andrea Domenico Praticò, Roberta Leonardi, Martino Ruggieri, Agata Polizzi

**Affiliations:** 1 Department of Medical Surgical Sciences and Advanced Technologies “GF Ingrassia”, University of Catania, 95123 Catania, Italy; alessioleoncini1995@gmail.com; 2 Department of Radiology, Ospedale Umberto I, ASP Siracusa, 96100 Siracusa, Italy; fazio.alessandro1993@gmail.com; 3 PhD Programme in Innovative Technologies in Biomedical Sciences, Kore University of Enna, 94100 Enna, Italy; 4 Unit of Pediatric Clinic, Department of Clinical and Experimental Medicine, University of Catania, 95123 Catania, Italy; lobiancomanuela@gmail.com (M.L.B.); leonardi.roberta@outlook.it (R.L.); mruggie@unict.it (M.R.); agata.polizzi1@unict.it (A.P.); 5 Department of Medicine and Surgery, University Kore of Enna, 94100 Enna, Italy; andrea.pratico@unikore.it

**Keywords:** CLIPPERS, pediatric neuroinflammation, magnetic resonance imaging, brainstem disorders, corticosteroid-responsive disease

## Abstract

**Highlights:**

**What are the main findings?**

- This systematic review summarizes the currently available evidence on the clinical, neuroradiological, cerebrospinal fluid, treatment, and outcome characteristics reported in pediatric CLIPPERS.
- The available evidence suggests that pediatric CLIPPERS may present with neuroimaging findings extending beyond the classic pontocerebellar pattern, including supratentorial and spinal cord involvement in selected cases, while generally maintaining a corticosteroid-responsive course.

**What are the implications of the main findings?**

- Awareness of the reported clinical and neuroimaging manifestations may facilitate earlier recognition of pediatric CLIPPERS and support timely diagnostic evaluation and immunosuppressive treatment in selected patients.
- Further prospective multicenter studies are needed to validate the proposed clinical and neuroimaging spectrum, refine diagnostic criteria, optimize long-term immunosuppressive strategies, and better define outcomes in pediatric CLIPPERS.

**Abstract:**

**Background:** Chronic lymphocytic inflammation with pontine perivascular enhancement responsive to steroids (CLIPPERS) is an uncommon inflammatory disorder of the central nervous system that is exceptionally rare during childhood. Although pontocerebellar punctate enhancement is considered its neuroradiological hallmark, the full spectrum of pediatric clinical and imaging manifestations remain poorly characterized because available evidence is limited to isolated case reports and small case series. We conducted a systematic review to characterize the clinical presentation, neuroimaging phenotype, diagnostic challenges, therapeutic strategies, and outcomes of pediatric CLIPPERS. **Methods:** A systematic literature search was conducted in PubMed/MEDLINE and Scopus from database inception to December 2025, following PRISMA guidelines. Studies reporting patients younger than 18 years with a diagnosis of CLIPPERS were eligible. Clinical, neuroradiological, laboratory, histopathological, therapeutic, and outcome data were extracted using a standardized form. Diagnostic certainty was assessed according to the Tobin criteria, and methodological quality was evaluated using the Joanna Briggs Institute (JBI) critical appraisal tools. **Results:** Ten studies comprising 13 pediatric patients fulfilled the inclusion criteria. Median age at presentation was 13 years (range 3–16 years), with a slight male predominance. Ataxia was the most common presenting manifestation (84.6%), followed by cranial nerve involvement (61.5%) and diplopia (46.2%). Magnetic Resonance Imaging (MRI) consistently demonstrated brainstem involvement, predominantly affecting the pons (84.6%) and cerebellum (76.9%), while supratentorial lesions (46.2%), midbrain involvement (46.2%), and spinal cord abnormalities (23.1%) suggest that the neuroradiological phenotype may extend beyond the classic pontocerebellar pattern, although these findings were observed in a limited number of patients. Cerebrospinal fluid (CSF) findings were nonspecific, typically showing normal results or mild lymphocytic pleocytosis. Histopathological examination, when available, demonstrated characteristic perivascular T-cell-predominant inflammatory infiltrates. All patients received high-dose corticosteroids with initial clinical improvement; however, relapses occurred in 61.5% of cases, frequently during corticosteroid tapering, often requiring steroid-sparing immunosuppressive therapy. Based on the available evidence, we suggest a practical clinic-radiological framework to facilitate the evaluation of children presenting with suspected CLIPPERS. **Conclusions:** Pediatric CLIPPERS represents a rare but increasingly recognized neuroinflammatory disorder characterized by marked corticosteroid responsiveness and a wide range of neuroradiological phenotypes. Although pontocerebellar involvement remains the defining imaging feature, supratentorial and spinal cord lesions are not uncommon and should not exclude the diagnosis after careful consideration of alternative inflammatory, autoimmune, infectious, and neoplastic disorders. Given the high relapse rate and the limited quality of available evidence, prolonged follow-up and collaborative multicenter studies are needed to refine pediatric diagnostic criteria and optimize long-term management.

## 1. Introduction

Chronic Lymphocytic Inflammation with Pontine Perivascular Enhancement Responsive to Steroid (CLIPPERS) was first described in 2010 by Sean J. Pittock et al., who reported a series of eight patients sharing distinctive clinical, radiological and pathological features associated with a marked response to corticosteroid therapy [1]. Since its original description, CLIPPERS has been increasingly recognized as a rare inflammatory disorder of the central nervous system predominantly involving the brainstem and cerebellum. Clinically, CLIPPERS is characterized by a relapsing–remitting course and typically presents with subacute brainstem and cerebellar dysfunction. The most frequently reported manifestations include gait ataxia, dysarthria, diplopia, and cranial nerve palsies [2]. Additional neurological symptoms, such as sensory disturbances, long-tract signs, and cognitive or behavioral changes, may also occur depending on the extent of central nervous system involvement. Magnetic resonance imaging (MRI) represents the cornerstone of diagnosis and usually demonstrates a characteristic pattern of multiple punctate and curvilinear gadolinium-enhancing lesions predominantly involving the pons, often extending to the cerebellum, medulla, midbrain, cranial nerves, and spinal cord [2,3]. The typical “peppering of the pons” appearance on post-contrast MRI is considered a radiological hallmark of the disease. Neuropathological examination commonly reveals dense perivascular lymphocytic infiltrates with a predominance of CD4 positive T lymphocytes, supporting an immune-mediated inflammatory pathogenesis, as frequently happens in other neurological disorders [3,4,5,6,7,8,9]. In 2017, William O. Tobin et al. proposed diagnostic criteria integrating clinical, radiological, and neuropathological findings in order to improve diagnostic accuracy and distinguish CLIPPERS from its numerous mimics [3]. A prompt response to high-dose corticosteroid therapy is one of the defining features of the disease. However, relapses frequently occur during steroid tapering or withdrawal, often requiring prolonged immunosuppressive treatment to prevent neurological deterioration and cumulative disability [1,2,3,4,5,6,7,8,9,10]. Although more than one hundred cases of CLIPPERS have been reported in the literature, pediatric-onset disease remains exceptionally rare. Consequently, the clinical spectrum, neuroradiological features, long-term outcome, and optimal therapeutic strategies in children are still incompletely understood. Given the rarity of pediatric CLIPPERS and the growing recognition of atypical neuroradiological presentations, we performed a systematic review of the literature to better characterize clinical manifestations, neuroimaging findings, laboratory features, therapeutic approaches, and outcomes in pediatric patients. Particular emphasis was placed on defining the neuroimaging spectrum and identifying diagnostic challenges relevant to clinical practice.

## 2. Methods

### 2.1. Study Design

This study was conducted as a systematic review in accordance with the Preferred Reporting Items for Systematic Reviews and Meta-Analyses 2020 (PRISMA 2020) statement [11]. The review protocol was not prospectively registered. The completed PRISMA 2020 Checklist is provided as Supplementary Material (Supplementary Table S1). The absence of prospective protocol registration is acknowledged as a methodological limitation. Given the descriptive nature of this systematic review, which synthesized published pediatric case reports and case series to characterize the clinical spectrum of CLIPPERS rather than evaluate a specific intervention or comparative outcome, prospective protocol registration was not undertaken. The review aimed, in fact, to comprehensively summarize the demographic, clinical, neuroradiological, laboratory, therapeutic, and outcome characteristics of pediatric patients diagnosed with CLIPPERS.

### 2.2. Literature Search Strategy

A systematic literature search was conducted in PubMed/MEDLINE and Scopus from database inception to 30 December 2025. The search strategy was designed to identify all published reports describing pediatric patients with CLIPPERS. PubMed/MEDLINE and Scopus were selected because together they provide broad coverage of the biomedical and clinical literature, including the vast majority of peer-reviewed publications relevant to rare neurological disorders. The following search strings were used:

1. PubMed: (“CLIPPERS” OR “chronic lymphocytic inflammation with pontine perivascular enhancement responsive to steroids”) AND (“pediatric” OR “paediatric” OR “child” OR “children” OR “adolescent”).
2. Scopus: TITLE-ABS-KEY (“CLIPPERS” OR “chronic lymphocytic inflammation with pontine perivascular enhancement responsive to steroids”) AND TITLE-ABS-KEY (“pediatric” OR “paediatric” OR “child” OR “children” OR “adolescent”).

No restrictions regarding publication date were applied. Only articles published in English were considered eligible. To ensure comprehensive identification of relevant cases, the reference lists of all included studies were manually screened for additional eligible publications.

Studies were considered eligible if they fulfilled all of the following criteria:

1. Reported one or more pediatric patients (<18 years of age);
2. Described original clinical data (case reports, case series, or letters reporting original cases);
3. Reported diagnosis of CLIPPERS established by the original authors;
4. Sufficient clinical and neuroradiological information available for data extraction.

The following publication types were excluded:

1. Narrative reviews;
2. Systematic reviews;
3. Editorials;
4. Conference abstracts;
5. Commentaries;
6. Studies without original patient data;
7. Duplicate publications.

The literature search, title and abstract screening, full-text eligibility assessment, data extraction, and diagnostic reassessment were independently performed by A.L. and A.F. Any disagreements between the two reviewers were resolved through discussion until consensus was reached. As consensus was achieved in all cases, the involvement of a third reviewer was not required. The primary objective of this review was to summarize the currently available evidence regarding pediatric CLIPPERS, with particular focus on clinical presentation, MRI findings, laboratory and neuropathological characteristics, treatment strategies, and outcomes.

### 2.3. Diagnostic Classification

Whenever sufficient clinical, neuroradiological, and histopathological information was available, each case was independently reassessed by two reviewers according to the diagnostic criteria proposed by Tobin et al. Cases were classified as definite CLIPPERS, probable CLIPPERS, or CLIPPERS-like syndrome based exclusively on the data reported in the original publications. In instances where the available information did not allow an unequivocal classification or disagreements arose between reviewers, the cases were jointly re-evaluated through discussion until consensus was achieved. No additional assumptions beyond the published data were made during the reassessment process. Particular attention was paid to the presence of atypical clinical features, unusual MRI findings, pathological confirmation, and alternative diagnoses that could mimic CLIPPERS. Whenever sufficient information was available, each case was independently reassessed according to the diagnostic criteria proposed by Tobin et al.

### 2.4. Study Selection

**Figure 1:**
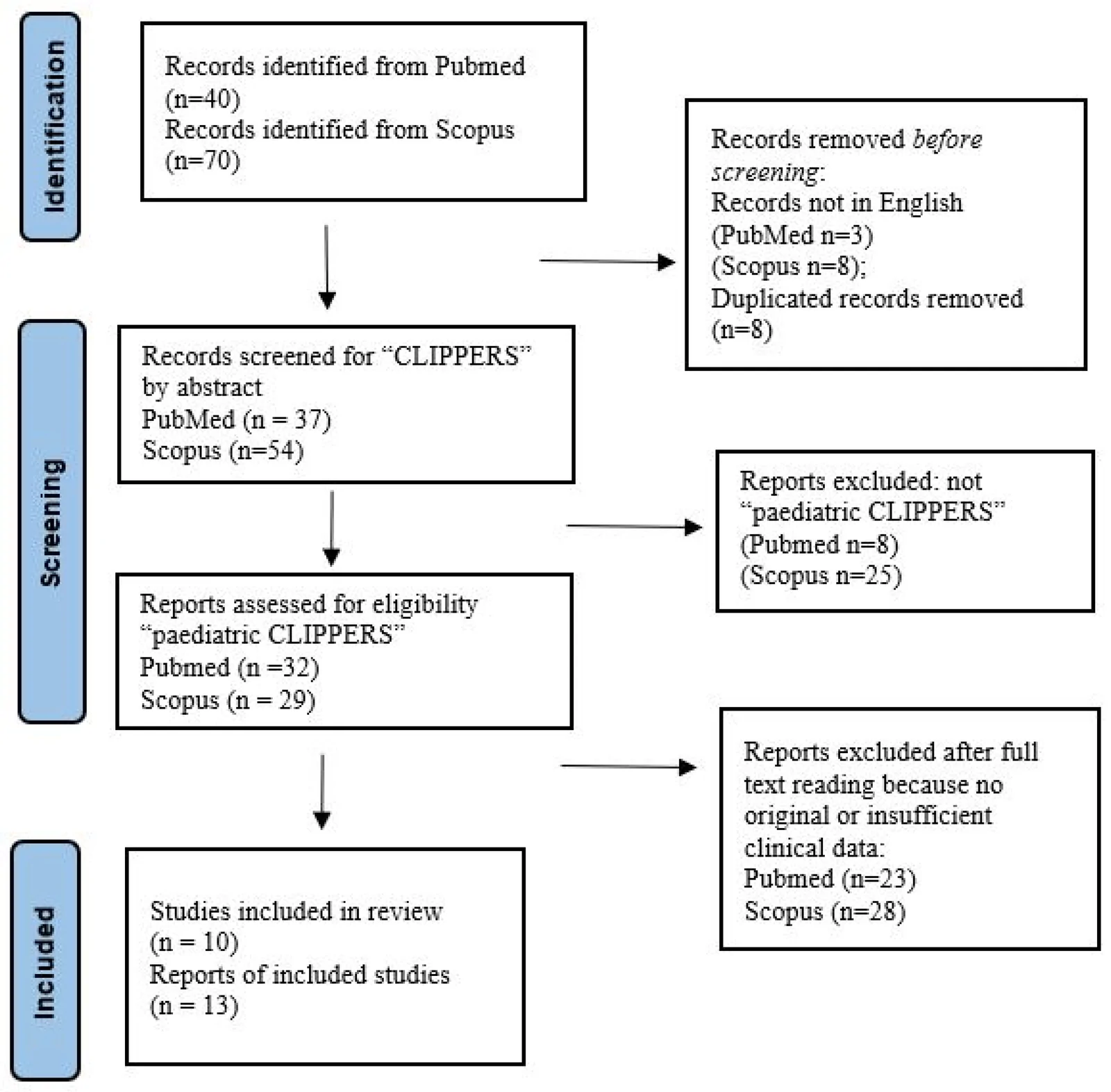
PRISMA flow chart of Literature research.

The literature search identified records through PubMed/MEDLINE and Scopus. After removal of duplicate records, titles and abstracts were screened for relevance. Full-text articles were subsequently assessed for eligibility according to the predefined inclusion and exclusion criteria. Only studies reporting pediatric patients with a diagnosis of CLIPPERS or CLIPPERS-like syndrome and providing sufficient clinical and neuroradiological information were included in the final qualitative synthesis. Overall, 40 articles related to CLIPPERS were initially identified through PubMed screening and 70 on Scopus. After title and abstract screening and full-text eligibility assessment, 10 studies reporting 13 pediatric patients were included in the final analysis [1,2,4,5,7,10,12,13,14,15]. The study selection process is summarized in Figure 1.

### 2.5. Quality Assessment

Methodological quality was assessed using the Joanna Briggs Institute (JBI) Critical Appraisal Checklist for Case Reports for individual case reports and the JBI Critical Appraisal Checklist for Case Series for case series. Item-level assessments were independently performed by two reviewers according to the published JBI guidance. The quality appraisal was used to support the interpretation of the available evidence and to identify potential methodological limitations of the included studies; however, no study was excluded based on its quality assessment.

### 2.6. Handling of Potential Diagnostic Mimics

Given the absence of a disease-specific biomarker and the recognized overlap between CLIPPERS and several inflammatory, demyelinating, neoplastic, infectious, and genetic disorders, particular attention was paid to the evaluation of alternative diagnoses in each included case. Clinical presentation, neuroimaging findings, cerebrospinal fluid (CSF) analysis, histopathological data, treatment response, and longitudinal follow-up were reviewed to determine whether alternative diagnoses had been adequately investigated and excluded by the original authors. Cases presenting atypical features, including extensive supratentorial involvement, longitudinal spinal cord lesions, optic pathway abnormalities, unusual enhancement patterns, poor steroid responsiveness, progressive disease despite immunotherapy, or subsequent diagnostic revision, were specifically highlighted.

## 3. Results

The literature search identified a total of 110 records, including 40 retrieved from PubMed/MEDLINE and 70 from Scopus. After removal of duplicate records, titles and abstracts were screened for eligibility. Full-text assessment was subsequently performed for potentially relevant articles. Following application of the predefined inclusion and exclusion criteria, 10 studies comprising 13 pediatric patients with CLIPPERS were included in the qualitative synthesis.

### 3.1. Study Selection and Characteristics of Included Studies

The included studies were published between 2010 and 2025 and consisted of eight single case reports, one case series including three patients, and one case series including two patients, for a total of 13 pediatric patients. Most publications originated from tertiary pediatric neurology centers and reported detailed clinical, neuroradiological, therapeutic, and follow-up information (Table 1).

### 3.2. Demographic Characteristics

Thirteen pediatric patients were included (7 males, 6 females; male-to-female ratio 1.2:1). Age at disease onset ranged from 3 to 16 years (median 13 years). Follow-up duration was highly variable, ranging from 8 months to 34 years, although long-term follow-up exceeding five years was available in several patients.

### 3.3. Quality Assessment

The methodological quality of the included studies was moderate according to the JBI Critical Appraisal Checklists. Most reports provided detailed descriptions of clinical presentation, neuroimaging findings, therapeutic interventions, and patient outcomes. The main limitations included the retrospective nature of the reports, variability in follow-up duration, incomplete reporting of diagnostic investigations, and the absence of standardized outcome measures (Table 2). The complete study-by-study JBI Critical Appraisal assessments, including the individual responses for each appraisal item, are provided as Supplementary Table S2.

### 3.4. Diagnostic Classification

Application of the Tobin diagnostic framework demonstrated that most reported pediatric cases fulfilled criteria for definite or probable CLIPPERS. All included cases were independently reassessed according to the diagnostic criteria proposed by Tobin et al. by two reviewers (A.L. and A.F.). Any discrepancies were resolved through discussion until consensus was reached. When published reports lacked sufficient clinical, radiological, or pathological information to allow complete application of the Tobin criteria, this was explicitly acknowledged during the reassessment. Cases fulfilling only CLIPPERS-like criteria were retained in the qualitative synthesis because they reflect the current spectrum of published pediatric reports, but their findings were interpreted with caution throughout the manuscript. Given the very limited number of available pediatric cases, no formal sensitivity analyses comparing definite/probable CLIPPERS with CLIPPERS-like cases were performed. A subset of patients exhibited atypical radiological or clinical features that broaden the recognized neuroimaging spectrum of pediatric CLIPPERS while also highlighting the importance of excluding alternative inflammatory, demyelinating, infectious, neoplastic, and genetic disorders (Table 3).

### 3.5. Pathogenesis

The pathogenesis of CLIPPERS remains incompletely understood. Current evidence supports an immune-mediated inflammatory process, mainly based on neuropathological findings demonstrating dense perivascular T-cell infiltrates within affected regions of the central nervous system. Given the characteristic anatomical distribution of lesions, Sean J. Pittock et al. hypothesized the presence of a specific antigenic epitope predominantly expressed within the pons and adjacent brainstem structures, potentially triggering a localized inflammatory response [1]. Subsequently, Taieb and colleagues emphasized the predominance of CD4-positive T lymphocytes in both biopsy specimens and CSF, further supporting the hypothesis of an autoimmune or antigen-driven immune reaction [4].

Another proposed mechanism involves a primary inflammatory process affecting small intra-axial venous structures, particularly within the brainstem, leading to secondary perivascular lymphocytic infiltration. In addition, Crowell et al. described an association between HLA-B27 positivity and CLIPPERS-like disease, raising the possibility of an underlying genetic or immune susceptibility [13,14]. However, the significance of this association remains uncertain and requires further investigation.

Increasing evidence also suggests a potential overlap between CLIPPERS and demyelinating disorders. A case reported by Symmonds et al. described neuropathological findings characterized by perivascular lympho-histiocytic infiltrates and perivenular demyelination resembling Acute Disseminated Encephalomyelitis (ADEM). Furthermore, associations with Myelin Oligodendrocyte Glycoprotein (MOG) antibody-associated disease have been increasingly recognized, supporting the hypothesis that CLIPPERS may represent, in selected cases, part of a broader inflammatory or demyelinating disease spectrum [15,16,17].

A further intriguing hypothesis concerns a possible genetic predisposition. Taleb et al. [18] described a series of adult patients with CLIPPERS-like syndrome, one-third of whom carried mutations associated with Hemophagocytic Lymphohistiocytosis (HLH). Based on these findings, the authors suggested considering genetic screening for HLH-related mutations in patients presenting with atypical or severe CLIPPERS phenotypes, particularly in cases characterized by poor response to corticosteroids, rapid progression, or systemic inflammatory manifestations [18]. This observation may have relevant therapeutic implications, as hematopoietic stem cell transplantation currently represents the possible curative treatment for primary HLH [18].

### 3.6. Clinical Features

The onset of CLIPPERS is typically subacute and progressive, reflecting the predominant involvement of the brainstem, cerebellum, cranial nerves, and, in some cases, the spinal cord. In the absence of adequate treatment, the disease usually follows a relapsing–remitting course, with cumulative neurological impairment related to recurrent inflammatory episodes [12,15,19]. The most frequently reported clinical manifestations include gait ataxia, dysarthria, diplopia, and cranial nerve palsies, reflecting the characteristic ponto-cerebellar localization of the inflammatory process. Oculomotor abnormalities, including nystagmus and impaired ocular movements, are also commonly observed. Additional neurological symptoms described in both adult and pediatric patients include headache, vertigo, tinnitus, fatigue, tremor, nausea, dysgeusia, sensory disturbances, and pyramidal signs with spastic para- or tetraparesis [1,2,3,10,12,13,14,15,16,17,18,19,20,21,22]. Spinal cord involvement may contribute to hyperreflexia, Babinski sign, sphincter dysfunction, and neurogenic bladder [20,23]. Cognitive and neuropsychiatric manifestations, including emotional lability, pathological crying or laughter, psychomotor slowing, and cognitive impairment, have also been reported, suggesting that CLIPPERS may extend beyond a purely brainstem disorder [1,15,17,19,20]. Ataxia represented the predominant presenting manifestation, occurring in 11/13 patients (84.6%). Cranial nerve involvement was documented in 8/13 patients (61.5%), most commonly affecting the abducens nerve. Diplopia occurred in 6/13 (46.2%), while dysarthria was reported in 5/13 (38.5%). Long-tract involvement, including lower-limb weakness, pyramidal signs, hyperreflexia, Babinski sign, or paraparesis, was observed in 5/13 patients (38.5%). Less frequent manifestations included headache (5/13; 38.5%), fever (2/13; 15.4%), nystagmus (5/13; 38.5%), encephalopathy (1/13; 7.7%), papilledema (1/13; 7.7%), tremor (2/13; 15.4%), neurogenic bladder (1/13; 7.7%), ptosis (1/13; 7.7%), seizures (1/13; 7.7%), vomiting (1/13; 7.7%) and vertigo (1/13; 7.7%) (Table 4).

Although the clinical presentation is often dominated by brainstem and cerebellar symptoms, the phenotypic spectrum appears broader than initially recognized. In pediatric patients, encephalopathy, seizures, papilledema, and multifocal neurological deficits have occasionally been described, potentially reflecting a more diffuse inflammatory involvement of the central nervous system [3,7,13,15]. A rapid and marked clinical response to high-dose corticosteroid therapy remains one of the hallmark features of the disease and represents an important diagnostic clue. Conversely, atypical clinical manifestations, poor steroid responsiveness, progressive neurological deterioration, or systemic inflammatory features should prompt reconsideration of alternative diagnoses and potential CLIPPERS mimics.

### 3.7. Neuroimaging

MRI plays a central role in the diagnosis of CLIPPERS, particularly during the early stages of the disease. The neuroradiological hallmark of CLIPPERS consists of multiple punctate and curvilinear gadolinium-enhancing lesions predominantly involving the pons, typically producing the characteristic “peppering of the pons” appearance on post-contrast T1-weighted images [15,17]. Lesions are usually bilateral and symmetrically distributed, with variable extension to adjacent structures including the cerebellar hemispheres, cerebellar peduncles, medulla, midbrain, cranial nerves, and spinal cord. Supratentorial involvement, affecting the basal ganglia, thalami, and cerebral white matter, has also been described, although less frequently. MRI abnormalities consistently involved the brainstem. Pontine lesions were identified in 11/13 patients (84.6%), confirming the pons as the principal anatomical site of involvement. Cerebellar abnormalities were observed in 10/13 patients (76.9%), whereas midbrain lesions occurred in 6/13 (46.2%). Medullary involvement was documented in 1/13 patients (7.7%). Supratentorial lesions were identified in 6/13 patients (46.2%), involving the cerebral white matter, thalami, basal ganglia, internal capsule, parieto-occipital regions, centrum semiovale, and cerebral cortex. Spinal cord abnormalities were reported in 3/13 patients (23.1%), involving the cervical and thoracic spinal cord. The characteristic punctate and curvilinear gadolinium-enhancing (“peppering”) pattern was present in 9/13 patients (69.2%). Less common neuroradiological findings included leptomeningeal enhancement (7.7%), optic chiasm involvement (7.7%), nodular enhancing lesions (7.7%), brainstem swelling (7.7%), and progressive brainstem atrophy (7.7%). The neuroradiological characteristics of published pediatric CLIPPERS cases are summarized in Table 5.

In most cases, lesion burden progressively decreases with increasing distance from the pons [1,14,15,16,17,18]. On T2-weighted and FLAIR sequences, the enhancing areas typically correspond to patchy hyperintense lesions without significant mass effect or necrosis. Importantly, the degree of T2 hyperintensity usually does not substantially exceed the extent of gadolinium enhancement, a feature that may help differentiate CLIPPERS from alternative inflammatory, neoplastic, or demyelinating disorders [3]. Although classically described as lacking mass effect, isolated cases with brainstem swelling or tumefactive-appearing lesions have been reported, particularly during relapses [24]. Therefore, the presence of mild mass effect should not automatically exclude the diagnosis of CLIPPERS. Rare cavitary T1 hypointense lesions (“black hole-like” lesions) have also been described in chronic or severe disease [25]. Longitudinal MRI follow-up is of particular importance, as radiological abnormalities may evolve over time and occasionally become more conspicuous during relapses than at disease onset. Progressive ponto-cerebellar and cerebral atrophy may occur in patients with recurrent inflammatory activity and has been associated with long-term neurological disability [17,25]. Advanced neuroradiological evaluation is essential to exclude disorders that may mimic CLIPPERS. Differential diagnosis includes inflammatory demyelinating diseases such as Multiple Sclerosis and MOG antibody-associated disease, infectious or granulomatous disorders such as neurosarcoidosis, vasculitis, autoimmune encephalitis, and neoplastic conditions including Primary Central Nervous System Lymphoma and glioma [16,17,24,26]. Notably, intracranial MR angiography, when performed, has generally failed to demonstrate imaging evidence of vasculitis [1]. To date, hypothalamic-pituitary involvement as well as meningeal and pachymeningeal lesions have not been consistently reported in CLIPPERS [26]. In Table 6 the main neuroradiological differential diagnoses of paediatric CLIPPERS are reported.

### 3.8. Neuroimaging Spectrum in Pediatric CLIPPERS

Neuroimaging findings represent the cornerstone of CLIPPERS diagnosis and remain the most distinctive feature of the syndrome. Pontine involvement represented the most frequent MRI finding (84.6%), followed by cerebellar lesions (76.9%), whereas supratentorial involvement (46.2%) was more common than previously appreciated. However, our analysis suggests that pediatric CLIPPERS may occasionally extend beyond the classic brainstem phenotype. However, these atypical neuroradiological findings were reported in only a limited number of published cases and should therefore be interpreted cautiously. Supratentorial abnormalities included involvement of the deep white matter, thalamus, basal ganglia, and internal capsule. Furthermore, spinal cord lesions were observed in 25.0% of cases, and optic pathway involvement was documented in one patient. The characteristic punctate gadolinium-enhancing “peppering” pattern remained the dominant radiological hallmark, being reported in two-thirds of patients. Nevertheless, atypical enhancement patterns, including nodular lesions and leptomeningeal enhancement, were also observed. Such findings broaden the recognized neuroimaging spectrum of pediatric CLIPPERS and may complicate differentiation from alternative inflammatory, demyelinating, infectious, and neoplastic disorders. Particularly noteworthy are the reports of extensive supratentorial disease, spinal cord involvement, brainstem swelling, and progressive atrophy. Although these features are intriguing, they were each reported in only one or a few patients and therefore cannot currently be regarded as established characteristics of pediatric CLIPPERS. Consequently, careful interpretation of MRI findings remains essential, especially when atypical radiological features are present. Overall, the currently available evidence suggests that pediatric CLIPPERS may not always be restricted to the pontocerebellar region. However, larger multicenter studies will be required to determine whether these atypical imaging findings represent true components of the disease spectrum or isolated observations. Rather, the disease appears capable of affecting multiple regions of the central nervous system while maintaining its characteristic steroid responsiveness and inflammatory radiological phenotype. The Neuroimaging Red Flags suggesting alternative diagnoses are reported in Table 7.

### 3.9. Laboratory Findings

Laboratory investigations in patients with suspected CLIPPERS are primarily aimed at excluding alternative inflammatory, infectious, neoplastic, and autoimmune disorders that may mimic the clinical and neuroradiological phenotype of the disease. To date, no disease-specific serum or CSF biomarker has been identified for CLIPPERS, making the diagnosis largely clinic-radiological and one of exclusion [1,3,16]. CSF analysis is frequently abnormal but usually nonspecific. Mild to moderate lymphocytic pleocytosis and elevated protein levels represent the most commonly reported findings, reflecting an underlying inflammatory process involving the central nervous system [4,17,19]. In several cases, CSF may remain entirely normal despite active disease, particularly during early stages or under corticosteroid treatment [1,10,12,13,14,15]. Oligoclonal bands (OCBs) have occasionally been detected, although their presence is typically transient and not considered a defining feature of CLIPPERS [2,16]. CSF analysis was reported for all included patients (13/13). Normal CSF findings were reported in 5/13 patients (38.5%). Pleocytosis represented the most frequent abnormality, occurring in 5/13 patients (38.5%), while elevated protein concentration was documented in 4/13 (30.8%). Oligoclonal bands were detected in 1/13 patients (7.7%). CSF findings of the 13 patients included in our review are reported in Table 8.

Brain biopsy was performed in 6/13 patients (46.2%). Histopathological examination consistently demonstrated predominantly perivascular lymphocytic inflammatory infiltrates with T-cell predominance and without evidence of necrotizing vasculitis or primary demyelination, supporting the diagnosis of CLIPPERS. One patient was subsequently diagnosed with Epstein–Barr virus-associated B-cell lymphoma during follow-up. Compared with Multiple Sclerosis, persistent intrathecal immunoglobulin synthesis appears less frequent and less pronounced. Neuropathological studies consistently demonstrate a predominantly T-cell-mediated inflammatory process. Histological examination usually reveals dense perivascular lymphocytic infiltrates involving both white and grey matter, with a predominance of CD3-positive and CD4-positive T lymphocytes, associated with variable macrophage activation [1,3,17,20]. Importantly, frank vasculitis, granulomatous inflammation, necrosis, and extensive primary demyelination are typically absent, findings that may help distinguish CLIPPERS from disorders such as Neurosarcoidosis, Primary angiitis of the central nervous system, or inflammatory demyelinating diseases [3,16]. Given the broad differential diagnosis, laboratory work-up should systematically include investigations for infectious, autoimmune, granulomatous, paraneoplastic, and lymphoproliferative disorders. Recommended evaluations include infectious serologies, autoimmune screening, vasculitis markers, paraneoplastic and neuronal antibodies, serum angiotensin-converting enzyme levels, and, when clinically indicated, haematological and oncological investigations [12,17,22]. Particular attention should be paid to disorders increasingly recognized as CLIPPERS mimics, including MOG antibody-associated disease, autoimmune encephalitis, Primary Central Nervous System Lymphoma, and HLH [18,27,28]. Recent evidence has further expanded the immunological spectrum potentially associated with CLIPPERS-like syndromes. Associations with anti-MOG antibodies have been reported in selected patients presenting with brainstem-predominant inflammatory disease [16,29]. Moreover, Taleb et al. identified mutations related to HLH in adult patients with CLIPPERS-like phenotypes, suggesting that genetic and immune dysregulation may contribute to at least a subset of cases [16]. These findings reinforce the importance of extended immunological and genetic investigations, particularly in paediatric patients with atypical presentations, aggressive disease course, systemic inflammatory manifestations, or poor response to corticosteroid therapy. Although brain biopsy is not routinely required in typical steroid-responsive cases fulfilling established diagnostic criteria, neuropathological examination should be strongly considered in patients with atypical MRI findings, progressive clinical deterioration, poor steroid responsiveness, or suspicion of neoplastic disease, especially Primary Central Nervous System Lymphoma [30,31,32,33,34].

### 3.10. Diagnosis

The diagnosis of CLIPPERS remains challenging because no disease-specific biomarker or pathognomonic histopathological feature has yet been identified. Consequently, CLIPPERS should currently be regarded as a clinic-radiological syndrome characterized by a distinctive combination of steroid-responsive brainstem dysfunction, typical MRI findings, and exclusion of alternative inflammatory, infectious, demyelinating, vascular, and neoplastic disorders [1,3,16]. In 2017, Tobin et al. proposed diagnostic criteria integrating clinical, neuroradiological, and neuropathological findings in order to improve diagnostic accuracy and reduce the risk of misdiagnosis [3]. According to these criteria, “definite CLIPPERS” requires compatible clinical and MRI findings associated with characteristic histopathological evidence, whereas “probable CLIPPERS” may be diagnosed in patients fulfilling clinical and radiological criteria after careful exclusion of alternative etiologies. From a neuroradiological perspective, the most characteristic MRI feature is the presence of multiple bilateral punctate and curvilinear gadolinium-enhancing lesions predominantly involving the pons and cerebellum, usually without significant mass effect or necrosis [1,3,17]. Marked clinical and radiological responsiveness to corticosteroids represents another major diagnostic clue. However, these findings are not entirely specific and may overlap with several inflammatory or neoplastic conditions [16,27]. Increasing evidence suggests that CLIPPERS may represent a heterogeneous neuroinflammatory syndrome rather than a single nosological entity. Indeed, a growing number of disorders have been reported to mimic the clinical and radiological phenotype of CLIPPERS, including MOG antibody-associated disease, Multiple Sclerosis, Primary Central Nervous System Lymphoma, glioma, Neurosarcoidosis, vasculitis, autoimmune encephalitis, infectious disorders, and HLH [22,28,29,30,31,32,33,34,35]. Particular caution is required in patients presenting with atypical clinical or neuroradiological features. Red flags include marked mass effect, necrosis, progressive lesion enlargement despite corticosteroid therapy, persistent diffusion restriction, prominent leptomeningeal involvement, systemic inflammatory manifestations, or poor steroid responsiveness [27,32]. In such cases, alternative diagnoses should be strongly reconsidered and histopathological confirmation may become necessary. Of particular relevance, several reports have documented the subsequent development of Primary Central Nervous System Lymphoma in patients initially diagnosed with CLIPPERS [33,34]. These observations have led some authors to hypothesize that, in selected cases, CLIPPERS may represent a pre-lymphomatous inflammatory state or an early immune-mediated manifestation of occult lymphoproliferative disease rather than a distinct inflammatory disorder [35]. Consequently, long-term clinical and neuroradiological follow-up is mandatory, especially in patients with recurrent relapses, atypical MRI evolution, or incomplete response to immunotherapy. In pediatric patients, the diagnostic process may be even more complex because of the broader differential diagnosis of inflammatory and demyelinating disorders in childhood. Conditions such as ADEM, MOG antibody-associated disease, autoimmune encephalitis, CNS vasculitis, and neoplastic disorders may closely resemble CLIPPERS at disease onset [22,31,32,33,34,35]. Therefore, a multidisciplinary approach integrating clinical, neuroradiological, laboratory, immunological, and, when required, neuropathological findings is essential to achieve an accurate diagnosis. Brain biopsy should not be routinely performed in typical steroid-responsive cases fulfilling established clinico-radiological criteria. Nevertheless, biopsy should be strongly considered in patients with atypical imaging findings, diagnostic uncertainty, progressive disease despite immunotherapy, or suspicion of malignancy, particularly lymphoma [25].

### 3.11. Diagnostic Mimics and Overlapping Syndromes

Several published pediatric cases exhibited clinical or neuroradiological features that overlapped with alternative neuroinflammatory disorders [20,21,22]. The principal differential diagnoses considered across studies included MOG antibody-associated disease (MOGAD), ADEM, neuromyelitis optica spectrum disorder (NMOSD), primary central nervous system vasculitis, neurosarcoidosis, central nervous system lymphoma, infectious encephalitis, and HLH-related neuroinflammation. In most cases, the diagnosis of CLIPPERS was supported by the characteristic punctate gadolinium-enhancing brainstem lesions and marked corticosteroid responsiveness. However, several patients displayed atypical radiological features, including supratentorial lesions, spinal cord involvement, optic pathway abnormalities, leptomeningeal enhancement, or nodular enhancement patterns, emphasizing the importance of careful differential diagnosis and long-term follow-up (Table 9).

### 3.12. Treatment and Outcome

High-dose corticosteroid therapy currently represents the first-line treatment for CLIPPERS and constitutes one of the defining features of the disease. Most patients presented rapid clinical and radiological improvement following intravenous methylprednisolone administration, typically associated with marked reduction of gadolinium-enhancing lesions on follow-up MRI [1,2,15,16,17,23]. However, despite the usually dramatic initial response, long-term disease control remains challenging. Relapses frequently occur during corticosteroid tapering or after treatment discontinuation, often leading to progressive neurological disability related to recurrent inflammatory activity [18,19,25]. For this reason, prolonged oral corticosteroid therapy and early introduction of steroid-sparing immunosuppressive agents are commonly required, particularly in relapsing or pediatric cases. Several immunomodulatory and immunosuppressive therapies have been used with variable success in both adult and pediatric patients. Reported steroid-sparing agents include methotrexate, azathioprine, cyclophosphamide, mycophenolate mofetil, hydroxychloroquine, and intravenous immunoglobulins [1,16,17,18,19,20]. Among these, methotrexate and azathioprine appear to be the most frequently employed maintenance therapies in published series, mainly because of their relative long-term tolerability and steroid-sparing effect. More recently, increasing attention has focused on B-cell-depleting therapies. Cipriani et al. reported successful long-term disease stabilization with rituximab in a patient with relapsing CLIPPERS, suggesting a possible role for targeted immunotherapy in selected cases [36]. Rituximab may be particularly useful in patients with aggressive relapsing disease, steroid dependence, or concern for overlap with autoimmune or lymphoproliferative disorders. In pediatric patients, treatment decisions are particularly challenging because of the rarity of the disease and the potential long-term complications associated with chronic immunosuppression. Prolonged corticosteroid exposure during childhood may result in significant adverse effects including growth impairment, osteoporosis, metabolic complications, hypertension, mood disturbances, and increased infection risk. Consequently, early introduction of steroid-sparing strategies should be carefully considered in children with recurrent disease activity. Although most reported pediatric patients initially responded favorably to corticosteroids, several cases developed relapses during dose reduction, requiring escalation to additional immunosuppressive therapies [5,13]. Importantly, delayed treatment initiation or recurrent relapses may contribute to irreversible neurological sequelae and progressive cerebellar or brainstem atrophy on longitudinal MRI follow-up [17,25]. Because CLIPPERS remains a diagnosis of exclusion and may occasionally represent an early manifestation of alternative inflammatory or neoplastic disorders, careful long-term clinical and neuroradiological monitoring is mandatory even in apparently steroid-responsive patients [22,25,33,34,35,36]. In particular, progressive MRI abnormalities despite immunotherapy, incomplete steroid responsiveness, or atypical clinical evolution should prompt reconsideration of the diagnosis and possible histopathological reassessment. To date, no standardized therapeutic protocol or evidence-based treatment guidelines are available for pediatric CLIPPERS and current management strategies are therefore largely based on individual case reports, small case series, and extrapolation from adult experience. All patients received high-dose intravenous methylprednisolone during the acute phase. Subsequent oral corticosteroid tapering was administered in nearly all cases. Because of disease relapses or corticosteroid dependence, steroid-sparing immunosuppressive therapy was frequently required. The most commonly employed agents included methotrexate, azathioprine, rituximab, mycophenolate mofetil, cyclophosphamide, natalizumab, infliximab, intravenous immunoglobulin, plasma exchange, and mercaptopurine. Clinical relapses occurred in 8/13 patients (61.5%), most frequently during corticosteroid tapering. Persistent neurological sequelae were observed in 4/13 patients (30.8%), including spastic paresis, hyperreflexia, neurogenic bladder, hemiplegia, bulbar dysfunction, and residual ataxia. Complete or near-complete clinical recovery following corticosteroid therapy was achieved in 5/13 patients (38.5%). Two patients (15.4%) died during follow-up. Treatment and outcomes of pediatric CLIPPERS are reported in Table 10.

## 4. Discussion

CLIPPERS represents a rare and still incompletely understood neuroinflammatory syndrome characterized by a distinctive combination of steroid-responsive brainstem dysfunction and punctate gadolinium-enhancing lesions predominantly involving the pons and cerebellum [1]. The application of the Tobin diagnostic criteria provides a structured framework for interpreting the limited pediatric literature. Although most reported cases fulfilled criteria for probable or definite CLIPPERS, several patients displayed atypical neuroradiological findings, reinforcing the need for careful differential diagnosis and long-term follow-up. Although the disorder has been increasingly recognized in adults over the last decade, paediatric-onset CLIPPERS remains exceptionally rare, with only a limited number of reported cases currently available in the literature (Table 11). The interpretation of these findings should be viewed in light of the very limited number of published pediatric cases. Consequently, several atypical neuroradiological features identified in this review should be considered hypothesis-generating observations rather than definitive characteristics of pediatric CLIPPERS. The present systematic review provides the most comprehensive synthesis to date of the neuroradiological characteristics of pediatric CLIPPERS. While punctate pontocerebellar enhancement remains the imaging hallmark of the disease, our analysis suggests that the MRI spectrum extends considerably beyond the classical infratentorial distribution. Nearly half of the included patients exhibited supratentorial abnormalities involving the cerebral white matter, thalami, basal ganglia, internal capsule, or cerebral cortex, whereas spinal cord involvement was documented in a smaller but clinically relevant proportion of cases. Additional atypical findings, including optic pathway involvement, leptomeningeal enhancement, nodular lesions, brainstem swelling, and progressive atrophy, further emphasize that pediatric CLIPPERS should not be regarded as a disorder exclusively confined to the pons. These observations support a broader neuroradiological phenotype and reinforce the need for comprehensive MRI evaluation of the entire neuraxis at presentation and during follow-up. First, the disease appears to exhibit substantial clinical and neuroradiological heterogeneity in children. While the classical adult phenotype is predominantly characterized by ponto-cerebellar involvement, several pediatric cases demonstrated more extensive supratentorial and spinal cord abnormalities [10,12,13,27,28,29,30,31,32]. In particular, the recently reported series by Sun et al. described multifocal “pepper-like” enhancing lesions extending beyond the brainstem to involve the bilateral cerebellum, parieto-occipital cortex, and centrum semiovale, further supporting the concept of a broader neuroanatomical spectrum in pediatric disease [15]. Pontocerebellar involvement remained the dominant neuroimaging pattern; however, supratentorial abnormalities were identified in 46.2% of pediatric cases, spinal cord involvement in 23.1%, and atypical features including optic chiasm, thalamic, basal ganglia, internal capsule, and leptomeningeal involvement were also observed. These findings support a broader neuroradiological spectrum than the classic pontocerebellar phenotype originally described in adult CLIPPERS. Another relevant observation emerging from the available literature is the potential overlap between CLIPPERS and inflammatory demyelinating disorders. Associations with MOG antibody-associated disease have been increasingly reported [26,27,28,29], while neuropathological studies demonstrated perivascular lymphohistiocytic infiltrates associated with perivenular demyelinating lesions resembling ADEM [16,29]. These findings suggest that, at least in a subset of patients, CLIPPERS may represent part of a broader autoimmune or demyelinating disease spectrum rather than a distinct nosological entity. Diagnosing CLIPPERS during childhood remains particularly challenging because several inflammatory, infectious, neoplastic, and autoimmune disorders may initially present with overlapping clinical manifestations and similar MRI findings. In contrast to adults, pediatric patients frequently undergo extensive investigations to exclude acquired demyelinating disorders, primary CNS vasculitis, autoimmune encephalitis, MOG antibody-associated disease, neuromyelitis optica spectrum disorder, glial fibrillary acidic protein astrocytopathy, HLH, CNS lymphoma, and systemic inflammatory diseases. The differential diagnosis of pediatric CLIPPERS remains particularly challenging. Several inflammatory, infectious, vascular, and neoplastic disorders may closely mimic both the clinical presentation and MRI appearance of the disease [26,27,28,29,30,31]. Among these, Primary Central Nervous System Lymphoma deserves special attention because multiple reports documented the later development of lymphoma in patients initially diagnosed with CLIPPERS [27,30]. This observation has led some authors to hypothesize that CLIPPERS may occasionally represent a pre-lymphomatous inflammatory condition or an early immune-mediated manifestation of occult lymphoproliferative disease rather than an isolated inflammatory syndrome. From a neuroradiological perspective, MRI remains the cornerstone of diagnosis and follow-up. The characteristic punctate and curvilinear gadolinium-enhancing lesions involving the pons are highly suggestive but not pathognomonic [1,3,15,17,30]. Importantly, the pediatric cases included in this review frequently demonstrated more diffuse lesion distribution than typically observed in adults, occasionally involving supratentorial white matter and longitudinally extensive spinal cord regions [1,15]. This broader radiological phenotype may partly explain the increased diagnostic complexity in children. A prompt response to corticosteroid therapy remains one of the defining features of CLIPPERS. Most pediatric patients showed rapid clinical and radiological improvement after high-dose steroid administration [1,17,23]. Nevertheless, relapses during steroid tapering were common, frequently requiring prolonged immunosuppressive treatment [18,19]. Long-term management therefore represents a major challenge, particularly in pediatric patients, in whom chronic corticosteroid exposure may lead to substantial treatment-related morbidity. Although methotrexate and azathioprine remain among the most commonly used steroid-sparing agents, increasing attention has recently focused on targeted immunotherapies such as rituximab [36]. However, evidence remains limited to isolated case reports and small series, and no standardized treatment protocols are currently available for pediatric CLIPPERS. Another important finding emerging from this review is the potential prognostic relevance of early recognition and treatment initiation. Recurrent inflammatory activity may contribute to irreversible neurological sequelae and progressive cerebellar or brainstem atrophy on longitudinal MRI studies [15,16,17,25]. Therefore, early diagnosis and prompt immunotherapy may be crucial to reduce cumulative neurological damage and long-term disability. Current evidence increasingly supports the concept that CLIPPERS should be regarded as a heterogeneous clinic-radiological neuroinflammatory syndrome requiring careful exclusion of alternative diagnoses and long-term multidisciplinary follow-up. The main demographic, clinical, neuroradiological, laboratory, therapeutic, and outcome characteristics of the pediatric CLIPPERS cases included in this review are summarized in Table 11. The diagnostic complexity of pediatric CLIPPERS is further highlighted by the report of a patient initially diagnosed with CLIPPERS who was subsequently found to have Epstein–Barr virus-driven B-cell lymphoma on repeat biopsy. This observation reinforces growing evidence that CLIPPERS may occasionally represent a radiological and clinical phenotype shared by distinct pathological processes rather than a single disease entity. Consequently, long-term surveillance and reconsideration of alternative diagnoses remain essential, particularly in patients with atypical imaging findings, recurrent relapses, or incomplete treatment responses. Of interest, the inclusion of three CLIPPERS-like cases and one patient who subsequently developed Epstein–Barr virus-driven B-cell lymphoma underscores the diagnostic complexity of pediatric inflammatory brainstem disorders. These cases highlight the importance of long-term clinical and radiological follow-up and reinforce those atypical presentations should prompt continued diagnostic vigilance. Consequently, the findings of the present review should be interpreted as descriptive observations rather than definitive evidence of the pediatric CLIPPERS phenotype. The findings of this review have several practical clinical implications. First, the currently available evidence consistently supports that pediatric CLIPPERS typically presents as a corticosteroid-responsive inflammatory disorder with characteristic punctate gadolinium-enhancing lesions predominantly involving the pons and cerebellum. Second, additional neuroimaging findings, including supratentorial involvement, spinal cord lesions, optic pathway abnormalities, leptomeningeal enhancement, nodular enhancement, brainstem swelling, and progressive atrophy, were reported only in isolated cases and should therefore be regarded as possible rather than established manifestations of pediatric CLIPPERS. Third, atypical clinical evolution, poor or incomplete corticosteroid responsiveness, progressive radiological abnormalities, systemic manifestations, or histopathological findings inconsistent with CLIPPERS should prompt reconsideration of alternative diagnoses, including neoplastic, infectious, autoimmune, or other inflammatory disorders. Finally, given the diagnostic complexity and the occurrence of CLIPPERS-like presentations that may later evolve into alternative pathological entities, long-term clinical and radiological follow-up with periodic diagnostic reassessment remains essential, particularly in children with atypical features or an unexpected disease course. This review has several limitations. First, the available evidence is limited to a very small number of published pediatric cases, most of which were single case reports, precluding robust statistical analyses and limiting the generalizability of the findings. Second, diagnostic reassessment according to the Tobin criteria relied exclusively on information reported in the original publications, which was occasionally incomplete. An additional limitation relates to the search strategy. To improve specificity, pediatric-related search terms were combined with disease-specific keywords during the electronic search. Although this approach was defined a priori, it may have reduced search sensitivity by failing to retrieve isolated reports in which patient age was not adequately represented in the title, abstract, or indexing terms. This potential source of selection bias should be considered when interpreting the findings. Finally, the review protocol was not prospectively registered. Although this does not affect the conduct of the review, the absence of prospective registration may reduce methodological transparency and should be considered when interpreting the findings.

## 5. Conclusions

Pediatric CLIPPERS remains an exceptionally rare inflammatory disorder characterized by marked corticosteroid responsiveness and a distinctive but evolving neuroradiological phenotype. This systematic review may indicate that, although pontocerebellar involvement remains the imaging hallmark, the disease frequently extends beyond the posterior fossa, involving supratentorial structures and, less commonly, the spinal cord and other atypical anatomical regions. These observations support a broader concept of the pediatric neuroimaging spectrum and emphasize the importance of comprehensive MRI evaluation of the entire neuraxis. Given the considerable overlap with inflammatory, autoimmune, infectious, and neoplastic disorders, pediatric CLIPPERS should remain a diagnosis of exclusion supported by careful clinico-radiological correlation and, when indicated, histopathological assessment. Finally, the high frequency of disease relapses highlights the need for prolonged follow-up and optimized steroid-sparing treatment strategies. Prospective multicenter collaborative studies are now required to validate pediatric-specific diagnostic criteria, identify reliable prognostic biomarkers, and establish evidence-based therapeutic recommendations.

## 6. Limits and Future Directions

This review has several limitations that should be acknowledged. First, the currently available evidence on pediatric CLIPPERS is extremely limited and is based almost exclusively on isolated case reports and small retrospective case series. Consequently, the small sample size restricts the possibility of drawing definitive conclusions regarding disease pathogenesis, prognosis, optimal therapeutic strategies, and long-term outcomes. Second, substantial heterogeneity was observed across published studies in terms of diagnostic criteria, neuroradiological evaluation, treatment approaches, and duration of follow-up. In several cases, incomplete clinical, laboratory, or imaging data further limited accurate comparison between patients. Moreover, given the evolving understanding of CLIPPERS over time, some previously reported cases may have represented alternative inflammatory, demyelinating, or lymphoproliferative disorders currently recognized as CLIPPERS mimics. Another important limitation is the absence of validated disease-specific biomarkers. The diagnosis of CLIPPERS therefore remains largely clinico-radiological and dependent on exclusion of alternative conditions, increasing the risk of diagnostic uncertainty and potential misclassification bias. In addition, publication bias likely favored the reporting of atypical, severe, or diagnostically challenging pediatric presentations. The present review has additional methodological limitations related to the literature search. First, the electronic search was restricted to PubMed/MEDLINE and Scopus. Although these databases provide broad coverage of the biomedical literature, relevant reports indexed exclusively in other databases may not have been identified. Second, only English-language publications were included. Consequently, eligible pediatric cases reported in other languages may have been missed, potentially limiting the completeness of the available evidence. These limitations should be considered when interpreting the findings. Finally, the review protocol was not prospectively registered. Although this does not affect the conduct of the review itself, the absence of prospective registration represents a methodological limitation because it may reduce transparency regarding the prespecified eligibility criteria, data extraction procedures, and synthesis decisions. This should be considered when interpreting the findings. Future studies should focus on the development of multicenter international registries in order to improve case collection, standardize diagnostic work-up, and better characterize the natural history of pediatric CLIPPERS. Advanced neuroradiological techniques, immunological profiling, neuropathological studies, and genetic investigations may further clarify whether CLIPPERS represents a distinct nosological entity or part of a broader neuroinflammatory disease spectrum. Particular attention should also be directed toward the relationship between CLIPPERS and other inflammatory disorders, especially MOG antibody-associated disease, as well as its possible association with Primary Central Nervous System Lymphoma and lymphoproliferative conditions. Finally, prospective collaborative studies will be essential to establish evidence-based treatment protocols, define optimal maintenance immunotherapy strategies, and identify predictors of relapse and long-term neurological outcome in pediatric patients.

## Figures and Tables

**Table 1:** Characteristics of the Included Studies.

Study	Year	Country	Study Design	No. of Patients	Follow-Up
Pittock et al. [1]	2010	USA	Case report	1	3.6 years
Salam et al. [2]	2014	Pakistan	Case report	1	1 year
Taieb et al. [4]	2012	France	Case report	1	34 years
Mathias et al. [5]	2015	USA	Case report	1	NR
Veerapandiyan et al. [10]	2017	USA	Case report	1	6 years
Nemani et al. [12]	2019	USA	Case report	1	7.2 years
Sa et al. [13]	2019	Portugal	Case series	3	1–14 years
Crowell et al. [14]	2019	USA	Case report	1	8 months
Lee et al. [7]	2022	Korea	Case report	1	19 months
Sun et al. [15]	2025	China	Case series	2	10 years/NR

**Table 2:** Quality Assessment of Included Studies.

Study	Design	JBI Score
Pittock et al. [1]	Case report	7/8
Taieb et al. [4]	Case report	7/8
Salam et al. [2]	Case report	7/8
Mathias et al. [5]	Case report	7/8
Veerapandiyan et al. [10]	Case report	7/8
Nemani et al. [12]	Case report	7/8
Crowell et al. [14]	Case report	7/8
Lee et al. [7]	Case report	6/8
Sa et al. [13]	Case series	8/10
Sun et al. [15]	Case series	8/10

**Table 3:** Diagnostic Classification of Included Paediatric CLIPPERS Cases According to Tobin Criteria.

Patient	Histopathology	Clinical Criteria	MRI Criteria	Classification
Pittock et al. [1]	No	Yes	Yes	Probable
Taieb et al. [4]	Yes	Yes	Yes	Definite
Salam et al. [2]	No	Yes	Yes	Probable
Mathias et al. [5]	No	Yes	Yes	Probable
Veerapandiyan et al. [10]	No	Yes	Yes	Probable
Nemani et al. [12]	Yes	Yes	Yes	Definite
Sa et al. Patient 1 [13]	Yes	Yes	Yes	Definite
Sa et al. Patient 2 [13]	Yes	Partial	Partial	CLIPPERS-like
Sa et al. Patient 3 [13]	Yes	Partial	Partial	CLIPPERS-like
Crowell et al. [14]	No	Partial	Partial	CLIPPERS-like
Sun et al. Patient 1 [15]	No	Yes	Yes	Probable
Sun et al. Patient 2 [15]	No	Yes	Yes	Probable
Lee et al. [7]	No	Yes	Yes	Probable

**Table 4:** Clinical features of published pediatric CLIPPERS cases.

Clinical Feature	Patients n (%)
Ataxia/gait disturbance	11 (84.6%)
Diplopia	6 (46.2%)
Cranial nerve palsy	8 (61.5%)
Nystagmus	5 (38.5%)
Dysarthria	5 (38.5%)
Limb weakness/paresis	5 (38.5%)
Headache	5 (38.5%)
Fever	2 (15.4%)
Visual disturbances	3 (23.1%)
Encephalopathy/altered mental status	1 (7.7%)
Tremor	2 (15.4%)
Vertigo	1 (7.7%)
Ptosis	1 (7.7%)
Seizures	1 (7.7%)
Vomiting	1 (7.7%)
Neurogenic bladder	1 (7.7%)
Papilledema	1 (7.7%)
Steroid responsiveness	13 (100%)

**Table 5:** Neuroradiological characteristics of published pediatric CLIPPERS cases.

MRI Finding	Number of Patients (%)
Brainstem involvement	13 (100)
Pontine lesions	11 (84.6)
Cerebellar lesions	10 (76.9)
Midbrain involvement	6 (46.2)
Medullary involvement	1 (7.7)
Supratentorial lesions	6 (46.2)
Spinal cord involvement	3 (23.1)
Cervical spinal cord	2 (15.4)
Thoracic spinal cord	2 (15.4)
Peppering enhancement	9 (69.2)
Curvilinear enhancement	2 (15.4)
Nodular enhancement	1 (7.7)
Leptomeningeal enhancement	1 (7.7)
Optic pathway involvement	1 (7.7)
Brainstem swelling	1 (7.7)
Brainstem atrophy	1 (7.7)

**Table 6:** Main neuroradiological differential diagnoses of pediatric CLIPPERS.

Disease	Typical MRI Findings	Distribution Pattern	Enhancement Pattern	Additional Distinguishing Features
CLIPPERS	Multiple punctate and curvilinear T2/FLAIR hyperintense lesions	Predominantly pons, cerebellum, cerebellar peduncles; may extend to medulla, midbrain, spinal cord	Characteristic punctate gadolinium enhancement (“peppering of the pons”)	Marked steroid responsiveness; relapsing–remitting course; usually minimal mass effect; CD4-predominant perivascular infiltrates
MOG antibody-associated disease	Large, poorly demarcated T2 hyperintense lesions; frequent bilateral involvement	Brainstem, cerebellum, deep white matter, optic pathways, spinal cord	Variable, often fluffy or patchy enhancement	Optic neuritis and longitudinally extensive transverse myelitis common; anti-MOG antibodies positive; overlap with ADEM phenotype in children
Multiple Sclerosis	Ovoid periventricular and juxtacortical lesions	Periventricular white matter, corpus callosum, infratentorial regions, spinal cord	Nodular or ring enhancement during active disease	Dawson’s fingers; dissemination in time and space; oligoclonal bands frequently positive
Primary Central Nervous System Lymphoma	Mass-like or infiltrative lesions with diffusion restriction	Deep periventricular white matter, basal ganglia, corpus callosum	Homogeneous or nodular enhancement	Progressive course; reduced steroid responsiveness over time; elevated perfusion restriction; biopsy often required
Neurosarcoidosis	Multifocal inflammatory lesions involving parenchyma and meninges	Basal meninges, hypothalamus, pituitary stalk, cranial nerves	Leptomeningeal and pachymeningeal enhancement frequent	Systemic sarcoidosis; elevated ACE levels; granulomatous inflammation
Primary angiitis of the central nervous system	Multifocal ischemic or inflammatory lesions	Cortical-subcortical regions, deep gray nuclei, brainstem	Variable enhancement	Vessel irregularities on angiography; stroke-like episodes; inflammatory CSF
Autoimmune GFAP astrocytopathy	Radial perivascular T2 hyperintensities	Periventricular white matter, brainstem, spinal cord	Linear radial perivascular enhancement	Meningoencephalitis-like presentation; GFAP-IgG positivity in CSF; frequent meningeal involvement
ADEM	Bilateral asymmetric large T2 hyperintense lesions	Subcortical/deep white matter, thalami, basal ganglia, brainstem	Variable patchy enhancement	Monophasic encephalopathy; post-infectious onset; diffuse demyelinating pattern

Abbreviations: ADEM: acute disseminated encephalomyelitis; ACE: angiotensin-converting enzyme; CD4: cluster of differentiation 4; CLIPPERS: chronic lymphocytic inflammation with pontine perivascular enhancement responsive to steroids; CSF: cerebrospinal fluid; FLAIR: fluid-attenuated inversion recovery; GFAP-IgG: glial fibrillary acidic protein immunoglobulin G; MRI, magnetic resonance imaging; MOG, myelin oligodendrocyte glycoprotein.

**Table 7:** Neuroimaging Red Flags Suggesting Alternative Diagnoses.

MRI Feature	Potential Alternative Diagnosis
Extensive supratentorial lesions	MOGAD, ADEM
Longitudinal spinal cord lesions	MOGAD, NMOSD
Mass effect	CNS lymphoma
Progressive enlargement despite steroids	Lymphoma
Leptomeningeal enhancement	Neurosarcoidosis, infection
Optic pathway involvement	MOGAD
Necrosis	Vasculitis, infection
Persistent MRI progression	CLIPPERS mimic

Abbreviations: ADEM: acute disseminated encephalomyelitis; CLIPPERS: chronic lymphocytic inflammation with pontine perivascular enhancement responsive to steroids; CNS: central nervous system; MOGAD: myelin oligodendrocyte glycoprotein antibody-associated disease; MRI: magnetic resonance imaging; NMOSD: neuromyelitis optica spectrum disorder.

**Table 8:** Cerebrospinal Fluid Findings in Paediatric CLIPPERS Cases.

CSF Feature	Patients n (%)
Pleocytosis	5 (38.5%)
Elevated protein	4 (30.8%)
Oligoclonal bands	1 (7.7%)
Normal CSF profile	5 (38.5%)
Acellular CSF	3 (23.1%)

**Table 9:** Potential Diagnostic Mimics of Paediatric CLIPPERS.

Mimic	Overlapping Features	Features Suggesting Alternative Diagnosis
MOGAD	Brainstem lesions, steroid responsiveness, spinal cord lesions	Optic neuritis, longitudinally extensive transverse myelitis, MOG-IgG positivity
ADEM	Multifocal MRI lesions, encephalopathy	Large bilateral white matter lesions, monophasic course
NMOSD	Brainstem involvement, myelitis	AQP4-IgG positivity, LETM, optic neuritis
CNS Vasculitis	Multifocal enhancing lesions	Vascular abnormalities, ischemic lesions
Neurosarcoidosis	Cranial neuropathies, leptomeningeal enhancement	Systemic sarcoidosis, granulomatous pathology
CNS Lymphoma	Steroid responsiveness, enhancing lesions	Progressive enlargement, mass effect, atypical biopsy
HLH-related neuroinflammation	Brainstem lesions, inflammatory CSF	Systemic inflammation, cytopenias, HLH genetic defects
Infectious encephalitis	Brainstem symptoms, CSF pleocytosis	Microbiological evidence, lack of sustained steroid response

Abbreviations: ADEM: acute disseminated encephalomyelitis; AQP4-IgG: aquaporin-4 immunoglobulin G; CNS: central nervous system; CSF: cerebrospinal fluid; HLH: hemophagocytic lymphohistiocytosis; LETM: longitudinally extensive transverse myelitis; MOG-IgG: myelin oligodendrocyte glycoprotein immunoglobulin G; MOGAD: myelin oligodendrocyte glycoprotein antibody-associated disease; MRI: magnetic resonance imaging; NMOSD: neuromyelitis optica spectrum disorder.

**Table 10:** Treatment and Outcomes of Paediatric CLIPPERS.

Treatment	n (%)
IV methylprednisolone	13 (100)
Oral corticosteroids	12 (92.3)
Methotrexate	4 (30.8)
Azathioprine	3 (23.1)
Rituximab	3 (23.1)
IVIG	3 (23.1)
Mycophenolate	1 (7.7)
Cyclophosphamide	1 (7.7)
Natalizumab	1 (7.7)
Infliximab	1 (7.7)
Plasma exchange	2 (15.4)
Mercaptopurine	1 (7.7)

Abbreviations: IV: intravenous; IVIG: intravenous immunoglobulin.

**Table 11:** Published Cases of Chronic Lymphocytic Inflammation with Pontine Perivascular Enhancement Responsive to Steroids (CLIPPERS) Reported in the Literature.

Author/Year	Pittock et al. 2010 [1]	Taieb et al. 2012 [4]	Salam et al. 2014 [2]	Mathias et al. 2015 [5]	Veerapandiyan et al. 2017 [10]	Nemani et al. 2019 [12]	Sa et al. 2019 [13]	Crowell et al. 2019 [14]	Lee et al. 2022 [7]	Sun et al. 2025 [15]
Sex	F	M	M	M	F	M	2M/1F	M	M	1M/1F
Age (year-old)	16	13	16	14	10	13	*Patient 1*: 3 *Patient 2*: 13 *Patient 3*: 14	15	10	*Patient 1*: 9 *Patient 2*: 14
Ethnicity	-	White	Pakistani	-	Latino	-	White	Afro-American	-	China
Follow-up period	3.6 years	34 years	1 year	-	6 years	7.2 years	*Patient 1*: 14 years *Patient 2*: 1 year *Patient 3*: 3.9 years	8 months	19 months	*Patient 1*: 10 years *Patient 2*: NA
Personal history	-	-	-	-	-	-	*Patient 3*: Slight cognitive impairment.	HLA-B27+, bilateral acute progressing to chronic anterior uveitis	Pancytopenia 3 years before (secondary to viral infection)	NA
Symptoms at onset/presentation	Ataxia, paraparesis, diplopia, vertical upbeat nystagmus	Ataxia, dysarthria, diplopia, nystagmus incoordination, psychomotor slowing and tetra-paresis	Headache, fever, seizures, nystagmus, dysarthria, ataxia, lower extremity weakness. 1 generalized tonic–clonic seizure	bilateral VI nerve palsy, left VII nerve palsy, horizontal and vertical nystagmus, ataxia, incoordination and dysarthria	lower limb weakness, ataxia, diplopia, bilateral VI palsy, horizontal nystagmus, hyperreflexia Babinski sign, neurogenic bladder	Ataxia, bilateral VI cranial nerve palsy, papilledema, unequal pupils, vertigo, fever, vomiting	*Patient 1*: ataxia, bilateral VI nerve palsies, drooling, tremor. *Patient 2*: ataxia and dysarthria. *Patient 3*: encephalopathy and cranial neuropathies.	Acute onset of headaches, left upper eyelid ptosis, binocular diplopia.	Progressive headache, diplopia, and unsteady gait, ataxia, broad-based gait, dysdiadochokinesia, intentional tremors	Weakness in the lower limbs, unsteady gait, and visual disturbances, dizziness, headaches, and speech disorders.
MRI findings at onset	Punctuate and curvilinear enhancement peppering pons, cerebellum, midbrain. Cervical and thoracic spinal cord involvement. Less numerous and smaller lesions as distance from the pons increased	Contrast enhancing lesions in supratentorial area and spinal cord, with brainstem swelling and atrophy	Hyperintensity in the brainstem and cerebellar hemispheres with symmetric punctiform gadolinium enhancement ‘peppering’ the pons and cerebellum	Patchy, peppered T2 hyperintensities with perivascular distribution in brainstem, mostly pons, and bilateral cerebellum. MRI spine was unremarkable.	Hyperintensities with enhancement in the midbrain, pons, medulla, cerebellar peduncles, optic chiasm, cervical and thoracic spinal cord	Hyperintensity on T2 sequences in subcortical and deep white matter over parieto-occipital and bilateral cerebellar region	*Patient 1*: Bilateral punctuate pattern of contrast enhancement of the dorsal pons, thalamus, and cerebellum *Patient 2*: bilateral nodular lesions with contrast enhancement in the midbrain, brainstem, basal ganglia, and posterior limb of the internal capsule. *Patient 3*: T2 hyperintense signal in the left middle cerebellar peduncle and dorsal pons.	T2 hyperintense lesions in the right midbrain, superior colliculus, cerebellar peduncles and vermis, and leptomeningeal enhancement along the vermian foliae.	Signal abnormalities of punctate and curvilinear-pattern enhancement involving the perivascular space in the cerebellum and pons, with cranial and caudal extension on T2 and post-gadolinium image	Multifocal “pepper-like” enhancement involving the brainstem, bilateral cerebellum, parieto-occipital cortex, and centrum semiovale
CSF	Normal	Positive OCBs	Pleocytosis (90% lymphocytes), elevated protein (59 mg/dL)	Normal	Pleocytosis (51 leukocytes/lL, lymphocytic predominance)	Pleocytosis with 90% lymphocytes and normal protein, sugar, and lactate	*Patients 1,2,3*: acellular, normal protein, no OCBs, and normal lactate. *Patient 2*: persistent slightly elevated proteins.	Opening pressure of 26 cm H_2_O. Glucose 61 mg/dL, protein 80 mg/dL. Lymphohistiocytic pleocytosis.	Normal	Mildly elevated protein levels, lymphocytic pleocytosis, and discrete increases in immunoglobulins
Biopsy	Not performed	Parenchymal and peri- vascular inflammatory infiltrates. No demyelination, granulomatous inflammatory, necrotizing vasculitis pattern. Infiltrates predominantly composed of CD3 T cells.	Not performed	Not performed	Not specific perivascular T-lymphocyte infiltration	Lymphoplasmacytic leptomeningeal infiltrate, perivascular aggregation. No vasculitis/necrosis. Decreasing gradient of inflammatory infiltrate from the pial surface to the white matter parenchyma. Dystrophic calcification in cerebellar white matter	*Patient 1*: perivascular lymphocyte and macrophage infiltrates. CD4 T-cell predominance; no vasculitis or demyelination. No malignancy. *Patient 2*: Chronic inflammation: predominant T-cell and histiocyte. Second biopsy: Epstein–Barr virus driven B-cell lymphoma. *Patient 3*: non-specific inflammation	Not performed	Not performed	NA
Treatment (acute attack/first presentation)	1 g IVMP daily for 5 days with oral prednisone taper for 2 weeks.	IVMP—(not received long-term corticosteroid oral therapy).	IVMP followed by oral prednisone.	1 g IVMP for 5 days followed by tapering doses of oral prednisone	IVMP followed by tapering doses of oral prednisone	IVMP (30 mg/kg/day), followed by tapering doses of oral steroids	*Patients 1,2*: IVMP followed by tapering doses of oral prednisolone. *Patient 3*: IVMP + IVIG	IVMP followed by tapering doses of oral prednisone	IVMP (30 mg/kg/day), followed by tapering doses of oral steroids	*Patient 1*: prednisone + mercaptopurine. *Patient 2*: prednisone
Relapses	Yes, following glucocorticosteroid taper.	Relapsed 12 times	Relapse with azathioprine, no relapse on oral corticosteroid, methotrexate	-	Relapses with oral corticosteroid, methotrexate and rituximab	Asymptomatic for 6 years.	*Patients 1,2,3*: Multiple relapses with tapering steroids	One month into the taper, anterior uveitis flare.	No relapse	No relapse
Maintenance treatment/steroids-sparing agents	Oral prednisone, Azathioprine	One 4wk cycle of rituximab	Azathioprine replaced by methotrexate	-	Methotrexate and Rituximab	IVMP followed by 4 weekly injections of rituximab. Methotrexate 10 mg/m^2^/wk	*Patient 1*: Azathioprine, natalizumab, and IVIG because of side effects of steroids *Patient 2*: Mycophenolate mofetil as steroid-sparing agent. IVIG, PLEX, and infliximab *Patient 3*: Relapses treated with azathioprine, cyclophosphamide and PLEX.	Subcutaneous methotrexate 25 mg	Oral prednisone	Prednisone
EDSS/outcome	Complete resolution with steroids. Azathioprine did not control recurrence.	After multiple relapses, Final EDSS score 7	Complete resolution	-	Spastic paresis, hyperreflexia and neurogenic bladder	EDSS 5.5	*Patients 1,3*: died *Patient 2*: hemiplegia, ataxia, bulbar dysfunction	Minimal residual MRI signs of disease	Complete resolution with steroids	Improvement of symptoms and MRI

Abbreviations: CD3: cluster of differentiation 3; CD4: cluster of differentiation 4; CSF: cerebrospinal fluid; EDSS: Expanded Disability Status Scale; H_2_O: water; HLA-B27: human leukocyte antigen B27; IVIG: intravenous immunoglobulin; IVMP: intravenous methylprednisolone; MRI: magnetic resonance imaging; NA: not available; OCBs: oligoclonal bands; PLEX: plasma exchange.

## Data Availability

All data analysed during this study are included within the article and its Supplementary Material.

## Supplementary Materials

The following supporting information can be downloaded at https://www.mdpi.com/article/10.3390/brainsci16080790/s1: Table S1: PRISMA 2020 Checklist; Table S2: Quality assessment of the included case reports and case series according to the Joanna Briggs Institute (JBI) Critical Appraisal Checklists.
