# Supplementary material for: Pediatric CLIPPERS: Expanding the Neuroimaging Spectrum of a Rare Steroid-Responsive Neuroinflammatory Syndrome—A Systematic Review
Source: Brain Sci. 2026 Jul 27;16(8):790. doi: 10.3390/brainsci16080790 (PMC13510634; doi:10.3390/brainsci16080790)
Supplement: Supplementary file 1 [file brainsci-16-00790-s001.zip › Table S2.pdf]

| Study | Demographics | Timeline | Clinical condition | Diagnostic methods | Intervention | Post-intervention condition | Adverse events | Takeaway lessons | Score |
|---|---|---|---|---|---|---|---|---|---|
| Pittock et al. [1] | ✓ | ✓ | ✓ | ✓ | ✓ | ✓ | ✗ | ✓ | 7/8 |
| Taieb et al. [4] | ✓ | ✓ | ✓ | ✓ | ✓ | ✓ | ✗ | ✓ | 7/8 |
| Salam et al. [2] | ✓ | ✓ | ✓ | ✓ | ✓ | ✓ | ✗ | ✓ | 7/8 |
| Mathias et al. [5] | ✓ | ✓ | ✓ | ✓ | ✓ | ✓ | ✗ | ✓ | 7/8 |
| Veerapandiyan et al. [10] | ✓ | ✓ | ✓ | ✓ | ✓ | ✓ | ✗ | ✓ | 7/8 |
| Nemani et al. [11] | ✓ | ✓ | ✓ | ✓ | ✓ | ✓ | ✗ | ✓ | 7/8 |
| Crowell et al. [13] | ✓ | ✓ | ✓ | ✓ | ✓ | ✓ | ✗ | ✓ | 7/8 |
| Lee et al. [7] | ✓ | ✓ | ✓ | ✗ | ✓ | ✓ | ✗ | ✓ | 6/8 |
| Sa et al. [12]* | ✓ | ✓ | ✓ | ✓ | ✓ | ✓ | ✗ | ✓ | 8/10 |
| Sun et al. [14]* | ✓ | ✓ | ✓ | ✓ | ✓ | ✓ | ✗ | ✓ | 8/10 |

**Abbreviations:** ✓ = criterion fulfilled; ✗ = criterion not fulfilled. * *Quality assessment performed using the JBI Critical Appraisal Checklist for Case Series.*

**Table S2**. Case reports were assessed using the Joanna Briggs Institute (JBI) Critical Appraisal Checklist for Case Reports (8 items), whereas case series were evaluated using the JBI Critical Appraisal Checklist for Case Series (10 items).
